# Unearthing new learning opportunities: adapting and innovating through the ‘Antibiotics under our feet’ citizen science project in Scotland during COVID-19

**DOI:** 10.1099/acmi.0.000710.v3

**Published:** 2024-06-19

**Authors:** Rebecca Mary Cornwell, Kirsty Ross, Caius Gibeily, Isobel Guthrie, Pak Hei Li, Laurence Taylor Seeley, Yaxuan Kong, Ava True, Arun Barnes, Emma Nimmo, Gloriya Len, Ioana Oprea, Boyang Lin, Aswin Sasi, Vicky Chu, Chloe Davidson, Daniil Ulasavets, Grace Renouf-Bilanski, Maria Dmitrieva, Yana Leung, Ziying Ye, Sasha Brown, Meghna Vaidya, Jenna Hynes, Catherine Mullner, Priyansha Agarwal, Paul Johnston, Charlotte Thorley, Clarissa Melo Czekster

**Affiliations:** 1 School of Biology, University of St Andrews, North Haugh, St Andrews, KY16 9ST, UK; 2 School of Computer Science, University of St Andrews, North Haugh, St Andrews, KY16 9SX, UK; 3 College Gate, University of St Andrews, St Andrews, KY16 9AJ, UK; 4 School of Physics and Astronomy, University of St Andrews, North Haugh, St Andrews, KY16 9SS, UK; 5 School of Medicine, University of St Andrews, North Haugh, St Andrews, KY16 9TF, UK; 6 Independent researcher

**Keywords:** antibiotics, citizen science, microbiology, public engagement, school outreach

## Abstract

‘Antibiotics under our feet’ is a Scottish citizen science project that aimed to raise science capital in primary school learners and their teachers through measurement of microbial diversity in urban soil samples in the search for novel antimicrobial compounds. Resistance to antibiotics is rising, posing a global threat to human health. Furthermore, science, technology, engineering and mathematics (STEM) skills are in crisis, jeopardising our capacity to mobilise as a society to fight antimicrobial resistance (AMR). Originally conceived as a response to the AMR and STEM emergencies, our project was hit by the unprecedented challenge of engaging with schools during the COVID-19 pandemic. We describe how we adapted our project to enable remote participation from primary schools and youth groups, utilising COVID-19 response initiatives as opportunities for multi-level co-creation of resources with learners in primary, secondary, and higher education. We produced portable kit boxes for soil sample collection with learning activities and videos linked to the Scottish Curriculum for Excellence. We also addressed glaring project specific content gaps relating to microbiology on English and Simple English Wikipedia. Our hybrid model of working extended our geographical reach and broadened inclusion. We present here the inception, implementation, digital resource outputs, and discussion of pedagogical aspects of ‘Antibiotics under our feet’. Our strategies and insights are applicable post-pandemic for educators to develop STEM skills using soil, microbes, and antibiotics as a theme.

## Data Summary

To support others in replicating our work, all digital resources have been made available under a Creative Commons ShareAlike 4.0 licence on the Figshare platform. Validated digital resources can be downloaded from https://doi.org/10.6084/m9.figshare.c.6337403.v4. We have uploaded the sequencing data obtained by our project to NCBI Sequence Read Archive (SRA) database https://www.ncbi.nlm.nih.gov/sra with BioProject accession PRJNA1064560. The individual files can be accessed using SRA accessions SRX23211102-SRX23211117.

All data underpinning this publication is openly available from the University of St Andrews: https://doi.org/10.17630/b416d19a-a803-444d-853c-f5654b13b0f1.https://doi.org/10.17630/6be910a1-a747-4c6c-b048-22f601e5f48b. One figure is available in Supplementary File with the online version of this article.

## Introduction

Infections caused by bacteria resistant to antibiotic treatment due to antimicrobial resistance (AMR) [1] are a major global health threat of the twenty-first century [2]. The economic and human cost of AMR is devastating, increasing the length of hospital stays, health complications and likelihood of death [3]. Within education, science, technology, engineering and mathematics (STEM) skills are also in crisis [4]. Contributory factors to the STEM crisis include stereotypical perceptions of those working in STEM careers, a lack of diverse role-models, issues around gender equality, socioeconomic barriers to further and higher education, and challenges of providing an adequate grounding in the sciences, technology, and mathematics within the education system to name just a few. STEM skills include data analysis, problem solving, experimentation, collaborative working, creating and innovating [5]. National approaches, such as Scotland’s ‘STEM strategy for education and training’ [6], emphasise the urgent requirement for learners to develop STEM skills. Acquiring STEM skills is important and understanding their broader applications in society is vital.

### The environment as a source for new and old antibiotics

A gram of soil can hold over 10^9^ bacterial and archaeal cells [7], a vast reservoir for antibiotics (old and new). Antibiotics from human and livestock usage enter the environment through excretion, disposal, and runoff [8]. Chemicals like those from beauty products and electronic waste leech into the environment becoming stressors or resources for microorganisms. Studies have demonstrated links between environmental contamination (extensively reviewed in [9]), shifts in biodiversity and antimicrobial resistance [10]. The extent of this contamination and associated risks are not yet fully understood.

Tackling AMR needs a combination of strategies, and holistic engagement between scientists and society. Initiatives, including citizen science projects, have been proposed for discovering new antibiotics from soil [11], with urban soil a potential source [12]. Involving learners in the hunt for antibiotic compounds tackles both AMR and the STEM skills shortage.

### STEM skills development and citizen science

Public engagement with science is driven by factors including education, innovation, and inspiration [13]. Engagement through soil projects has had various motivators with all involving participants as active contributors to innovative research. Projects have invited the public to get hands-on with nature [14] and learn new skills [15] as well as engaging specific groups invested in soil health such as the farming community [16] and schools to teach core topics [17,18] and address STEM skills shortages [4].

AMR education was important when engaging the public through soil collection initiatives using apps [19,20]. DNA barcoding has been used in primary, secondary, community college and university settings [21,22] to investigate biodiversity. Large-scale community input was key to projects including environmental fungal surveillance [23,24], mapping soils and microbial diversity [25] and learners identifying metals in drinking water [26]. Although sharing similar long-term aspirations for increased STEM uptake, we focussed on a younger age group than the USA ‘Small World Initiative’ [27] and UK ‘Antibiotics Unearthed’ [19] projects, with more emphasis on participants exploring their local soil microbial environment than gaining experience with laboratory skills. We produced explanations on what happens in our lab to participants' samples and used social media for communication like the UK ‘Swab and Send’ [28] project. However, their successful low cost, high sample volume initiative leaned more towards centralised public engagement and gathering a library of diverse samples than creating bespoke learning materials to accompany sample collection and to help learners interpret scientific results. The ‘Swab and Send’ approach also relies on the ability of the organisms to be culturable on common laboratory media, typically plates or liquid culture. Not all soil microbes are culturable under these conditions and so isolating total environmental DNA enabled us to sidestep this common problem [29]. In common with the Australian ‘Soils for Science’ [30] project, we produced an interactive map, but we implemented that through our website not an app. In presenting our project here, we stress that we are sharing how our citizen science project, which may have been conceived with broadly similar increased antibiotic awareness and STEM educational goals to other citizen science projects, was continually reshaped by, and delivered responsively during the challenging uncertainty and varying restrictions of the COVID-19 pandemic.

Specifically relevant to our project context, a published analysis of fifty-five citizen science soil initiatives [31] highlighted important needs and found three common aspects: connections between soil and human health (with emphasis on food production and antibiotics); education for sustainable practices for a better future; and soil health and ecosystem conservation. Further, a review of citizen science soil toolkits reported suitable methods and kits for monitoring soil health indicators [32].

### Project context

Wellcome Trust funded research performed by our group at the University of St Andrews investigates the biosynthesis of natural products and other molecules with potential as novel antimicrobial compounds [33]. These molecules are thought to be produced by microbes to interfere with other microorganisms and their hosts [34]. Around 50 % of all antibiotics currently used in clinic are either natural products or molecules derived from natural products [35]. Additional funding support from a Scottish Public Engagement Network (ScotPEN) Scottish Wellcome Engagement Award (SWEA) gave rise to ‘Antibiotics under our feet’ (AUOF). Linked to Learning for Sustainability [36] and aligned with the Scottish Curriculum for Excellence [37], AUOF aimed to determine the microbial diversity in urban areas in Scotland and discover new natural products from soil microbes through citizen science.

### Initial setbacks

AUOF was initially planned to start in March 2020, lasting for 24 months. However, the COVID-19 pandemic led to school closures in Scotland from March 2020 [38]. Connecting with teachers and learners outwith the usual physical school environment presented a significant challenge [39]. The project start was therefore delayed until June 2020. Contingency funding and no-cost extensions enabled AUOF to continue until October 2023.

There is ongoing interest in studying pandemic responses to gain insight into education policies and practices [40]. This paper will chart our journey through a participatory research project during this unique period. We will outline the logic model underpinning the project and explain how learners' innovative contributions impacted on our project, leading to a variety of outputs informed by the synergy of bringing disparate groups together united by a common purpose.

## Methods

Our pilot study [41] conducted in four schools in Fife and Edinburgh Zoo during 2018–2019 highlighted development areas for discussing DNA and antibiotics with primary school audiences, and considerations for collecting school soil samples for downstream DNA analysis. Evaluation of the pilot guided the design of AUOF.

The initial funding application proposed travelling throughout Scotland conducting in-person workshops for learners and teachers, supporting them in sample collection. Unsurprisingly, COVID-19 and the subsequent lockdowns made this task impossible. It was necessary to pivot the project and create alternate pathways to achieve similar impact. The methods below reflect these changes.

### Logic model

#### Aim

AUOF aimed to raise science capital [42] in pupils from the Primary 5 to Primary 7 school year groups (learners aged 8–11 years old as contextualised in Table 1 that summarises audience statistics) and their teachers in Scottish schools. Science capital recognises the impact of STEM knowledge, attitudes, actions, and connections in shaping an individual’s relationship with science, technology, and mathematics in the broadest sense [43]. Concurrently, the project sought to acquire soil samples from urban locations as sources of environmental microbial DNA for research activities.

**Table 1.** Audience statistics (table adapted from 80)

Ages (years)	Educational stage	Key institutions	Learners engaged in our project*
2/3–5	Early learning and childcare	Early learning centres and nurseries	
5–12	Primary (P1-P7, compulsory)	Primary schools	EXPLORATHON (four sessions), participating schools (two schools) youth groups (three groups)
12–15	Lower Secondary (S1-S3, compulsory)	Secondary schools	S1 year group trialled activity
15–18	Upper Secondary (S4, compulsory; S5-6 optional) and Further Education	Secondary schools, Further Education Colleges	Work experience student (1), Developing the Young Workforce student (1), Nuffield Research Placement students (9)
17+	Higher Education (including undergraduate and postgraduate)	Higher Education Institutions	Undergraduates: interns (2), iGEM students (5), STEP students (20)Postgraduates: Masters (1)PhD (4)

* (* (nNumbers of learners actively generating and analysing data and co-creating materials)). Individual class sizes could be up to 33 learners and youth groups weekly attendance estimated between 10–20 participants present.

#### Objectives

We had three primary objectives:

1. To identify new sources of antibiotics by sequencing environmental DNA from schools across Scotland.
2. To build teacher confidence in STEM subjects, focusing on topical science.
3. To boost young people’s science capital by inclusion in a low barrier, citizen science project.

### Inputs

Various inputs were needed for project success. Wellcome Trust funding supported R. Cornwell as AUOF public engagement officer. C. Czekster provided project and line management support. K. Ross provided public engagement guidance, connection with non-school audiences, and assistance with archiving the project via online platforms such as Figshare. The team also received support from C. Thorley who provided independent summative evaluation of the project. Audiences engaged as part of the project included: young people and their teachers in schools; young people and their youth leaders outwith schools; community members; Wikipedia editors from Scotland and beyond (via the American Microbiology Society); undergraduate and postgraduate students, and researchers at the University of St Andrews. ‘Topical science’ in Scotland’s Curriculum for Excellence uses real-world contexts such as use of stem cells, cloning, climate change, genetically modified foods and the use of renewable energy sources for learning. Our project focused on the global antibiotic crisis. The project involved several consulting partners including the James Hutton Institute (JHI), University of Strathclyde, University of Glasgow, and Fife Council.

### How participation was enabled during the COVID-19 pandemic

Primary level learners engaged through direct responses to our call through the registration form on our project website and existing connections, including schools and groups participating in EXPLORATHON events via the University of St Andrews [30].

Recruitment of Secondary and Higher Education learners was facilitated by national and University of St Andrews initiatives. Nuffield Research Placements [44] for secondary pupils adopted a hybrid approach. Undergraduate students in the 2020 iGEM competition [45] integrated digital engagement for AUOF into their ‘integrated human practices’. St Andrews Flexible Fund internships (ST.A.F.F.) offered valuable paid work experience and the Summer Teams Enterprise Programme (STEP) enabled interdisciplinary teamwork when many undergraduate summer opportunities had lessened or were cancelled due to the pandemic. Additionally, postgraduate students and post-doctoral-, early career- and established researchers enhanced their public engagement skills through AUOF Professional Development opportunities.

### Activities

#### Website

From various options [23,24, 46], we selected a webform for remote soil data and photo uploads onto our map of the soil microbiome, creating a website with interactive map [www.antibioticsunderourfeet.ac.uk] as a central hub for our scientific information (communicated as infographics), activities, and results.

#### Physical kit boxes

Replacing onsite workshops, kit boxes were designed providing full equipment [47] and instructions necessary for teachers and youth workers to collect soil samples [48] and environmental data [49] with young people and return the samples to the University.

#### Learning materials

Scotland’s Curriculum for Excellence (CfE) [37] allows teacher flexibility over context when delivering Broad General Education over Early, First, Second, Third, and Fourth levels. These levels align with early-learning to lower-secondary education. We designed learning materials on soil, microbes, and DNA for Second Level learners. During planning [50], we brainstormed project activity areas using Education Scotland’s Benchmark information [51] looking also at First and Third levels as learners progress at differing paces. We identified applicable General Teaching Council for Scotland Professional Standards [52] to help teachers use their engagement with AUOF for Professional Learning [53].

Key CfE Experiences and Outcomes [54] underpinning our project are:

- *‘I can identify and classify examples of living things, past and present, to help me appreciate their diversity. I can relate physical and behavioural characteristics to their survival or extinction.’* SCN 2–01 a
- *‘Through research and discussion, I have an appreciation of the contribution that individuals are making to scientific discovery and invention and the impact this has made on society.’* SCN 2–20 a
- *‘I can report and comment on current scientific news items to develop my knowledge and understanding of topical science.’* SCN 2-20b

### Digital media content creation

iGEM undergraduates created instructional videos [55] about our soil sample processing explaining steps including collection, preparation, cultivation of microbes using lab footage, animations, and everyday items to clarify concepts like ‘What is DNA?’. With school visits impossible, St. Agatha’s Primary School learners submitted questions after online EXPLORATHON sessions [56]. University post-graduates and researchers responded with mobile phone videos. ST.A.F.F. undergraduate interns used these to create a ‘You asked, we answered’ video series [57,62] aligned with CfE leading to our YouTube channel launch enabling embedded videos on our site and participation in British Science Week’s Smashing Stereotypes Campaigns [63,64] in 2022 and 2023.

### Wikipedia editing

**Fig. 1.**
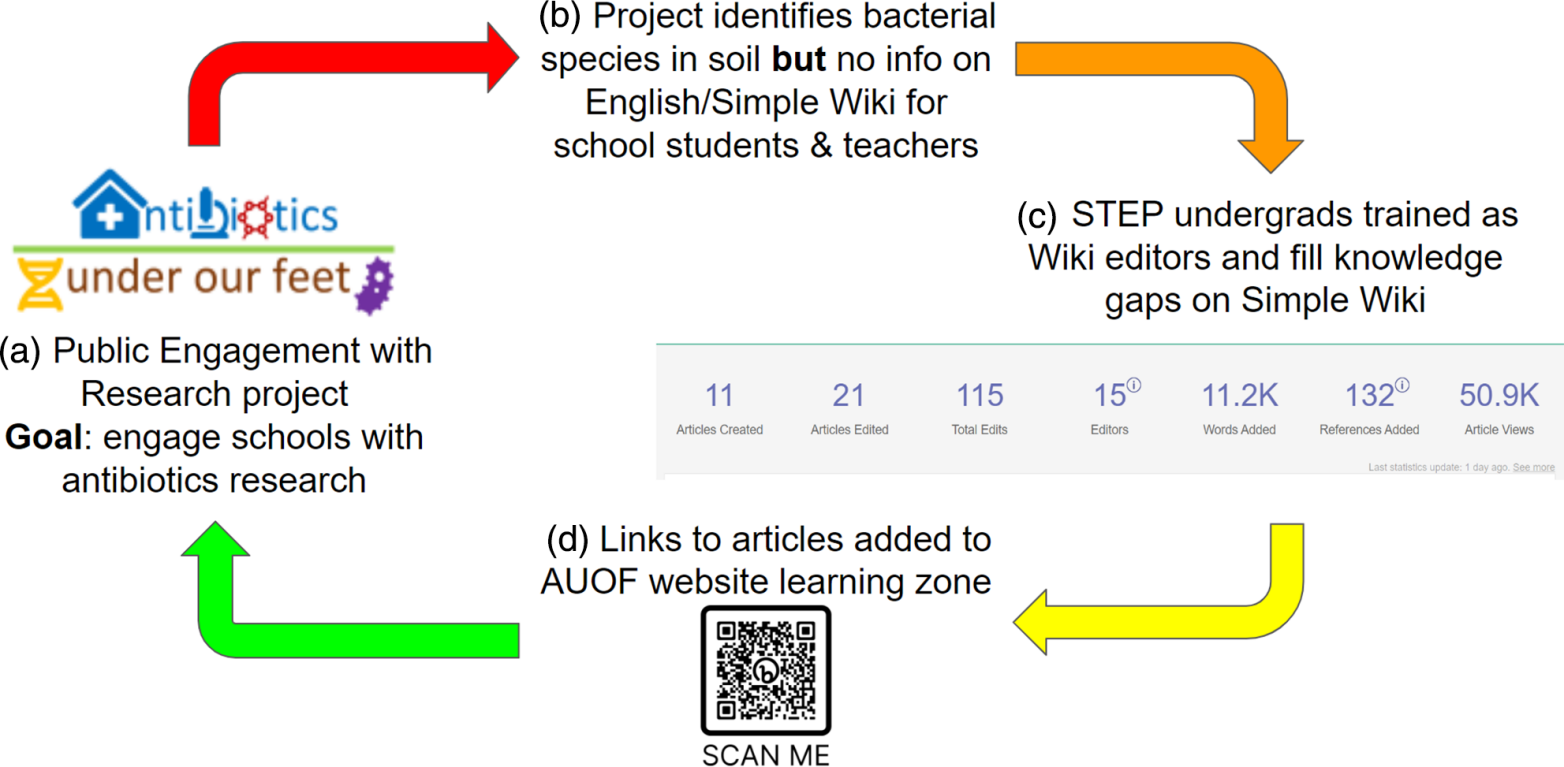
Flowchart for mechanism of action of Wikipedia editing. (**a**) Summary of project goal. (**b**) Challenge faced by teachers seeking additional information about the microbial DNA found in their soils. (**c**) Dashboard documenting progress of STEP students who stepped in to fill the knowledge gaps on English/Simple Wikipedia. (**d**) Links to articles were added to the AUOF website and could be reached via the QR code.

The flowchart depicted in Fig. 1 describes our mechanism for addressing content gaps relating to microbiology on English and Simple Wikipedia.

In 2022 and 2023, STEP recruited undergraduate students who received Wikipedia editor training from K. Ross. Their edits were monitored using an Outreach Dashboard set up as a standard edit-a-thon tracking changes on various Wiki projects like Wikipedia, WikiCommons, and WikiData during the approximately 6 week STEP period. Different language versions of Wikipedia were also tracked based on student interests, e.g. Simple English, Welsh, Mandarin. The dashboard permitted the creation of article lists for students to choose from. In 2022, the bacterial species were selected from a list derived from FERA [16]. In 2023, we used sequencing data about our collected samples to create a list focusing on the most prevalent genera and those present in all received samples. In addition, articles on human pathogens and antibiotics were identified for creation and improvement [65,66]. Following STEP 2023, students were asked to complete a service evaluation on their experience with the project.

We conducted a Wiki edit-a-thon in May 2023 with members of the American Society for Microbiology, with editors joining us from Singapore, Ghana, and the United States of America, to improve articles relating to soil microbes.

### Assumptions

Our assumptions in Table 2 below were directly checked with our audience members and based on strategic reports to the Scottish Government [67].

**Table 2.** Assumptions of our logic model

COVID-19 will not shutdown all Fife primary schools again in the next two academic years
Researchers lack knowledge of Curriculum for Excellence and how to adapt their resources so want to work with teachers to develop resources for schools
Teachers and learners want to engage with the University of St Andrews
Teachers and learners lack confidence in STEM subjects
Teachers and learners have low levels of science capital
Those involved have time to devote to the project
The local community want to contribute to this citizen science project

## Results

**Fig. 2.**
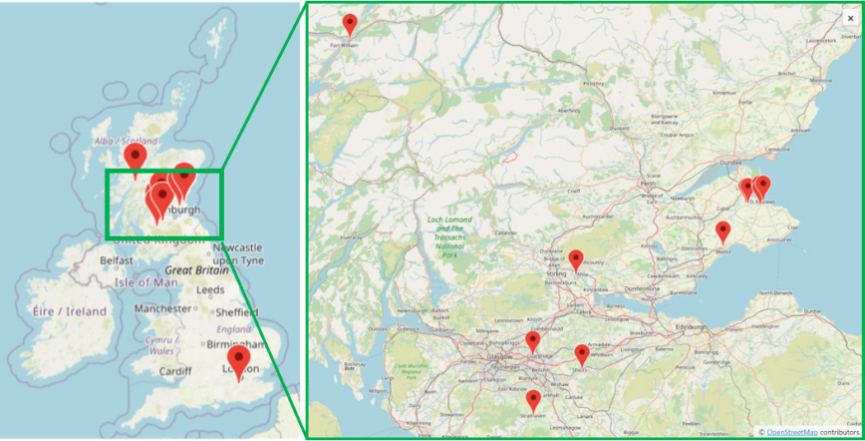
Map showing locations of soil samples collected and analysed for this project [map from OpenStreetMap Foundation under Creative Commons Attribution-ShareAlike 2.0 license].

Eleven soil samples were received from locations in the UK, mainly from schools and youth groups in Scotland (Fig. 2). A breakdown of our audience by educational stage is given in Table 1.

### Learning materials

**Fig. 3.**
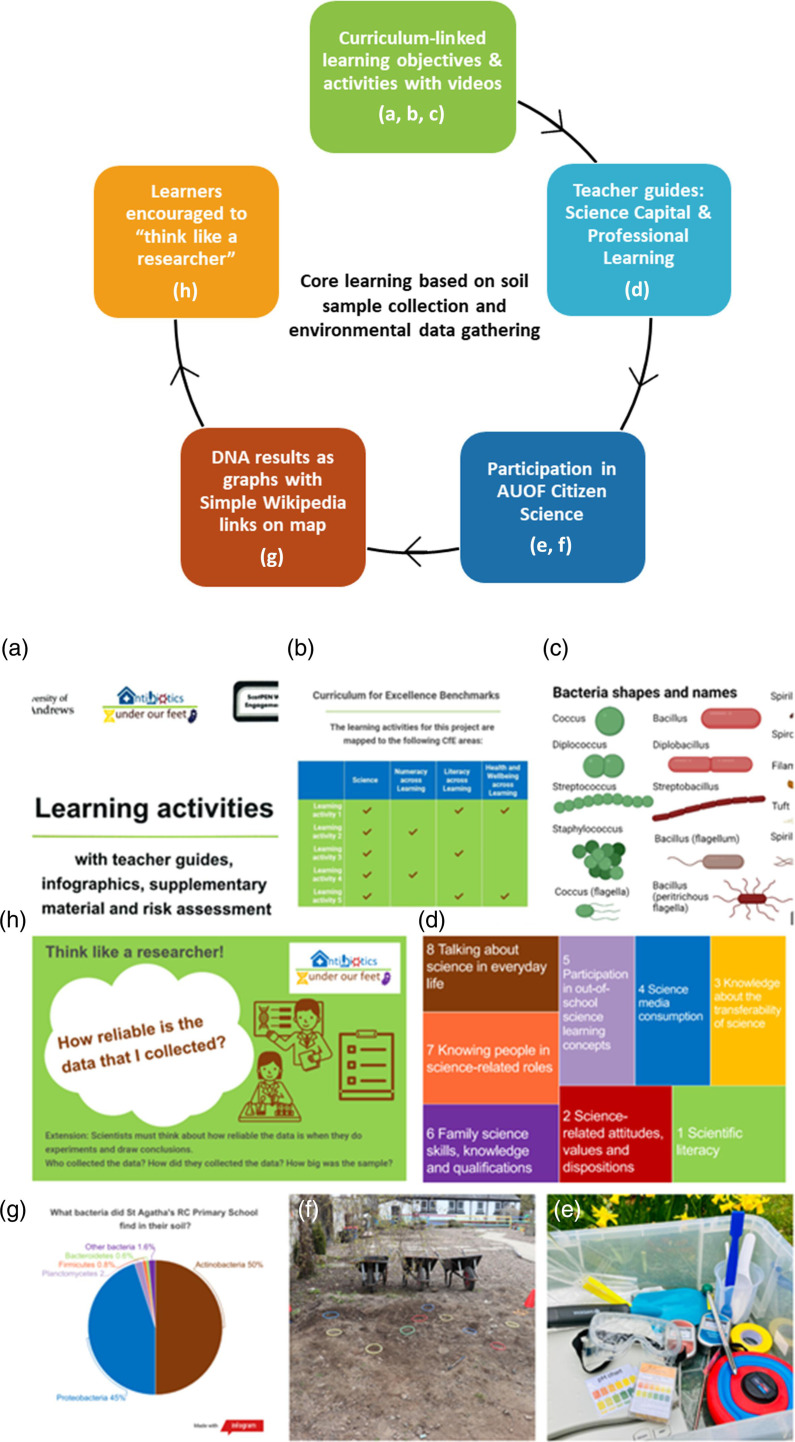
Core learning pathway (top) illustrated by snapshots: (a) learning activities with (**b**) objectives linked to CfE Sciences, Numeracy, Literacy and Health and Wellbeing across Learning [47,69,71] and resources including (**c**) ‘make a microbe’ activity with video (created with Biorender.com) [72]; (**d**) infographic from AUOF teacher guide to getting started with science capital [68]; (**e**) kit box contents and (**f**) soil sample collection site showing W-shaped transect marked out by Getting Better Together Shotts; (**g**) DNA sequencing results from St Agatha’s Primary School simplified as pie chart for Second level learners (created with Infogram.com) [69]; (**h**) example from ‘think like a researcher’ series [70].

Core learning (Fig. 3) included learning activities to provide a pathway through our project: ‘read our story’ [68], ‘collect a soil sample’ [69], ‘tell us about your patch of ground’ [70], ‘collect data about your patch of ground’ [71] and ‘what do we do with your soil samples’ [72]. Detailed teacher notes supported remote delivery. Additional learning resources were created, trialled, and posted online to help learners understand DNA sequencing and interpret our scientific results [73,75].

### Digital resources

Our AUOF articles on Figshare [76] have been viewed 5116 times and downloaded 1636 times from December 2022 to January 2024. The most downloaded article was about our pilot study followed by the ‘AUOF: bacteria pie charts learning activity’ [75].

Our initial website had over 3000 visits during 2021–2022. Of our website users, 34 were engaged users contributing to maps, forums, ideas, stories, questions, and polls. Two hundred and sixty-eight users downloaded documents, viewed content, and engaged with tools. Our new University of St Andrews WordPress blog website received 8258 visits from 2565 visitors in January to July 2023.

### Social media content creation

We used social media, mainly X (formerly Twitter), to create awareness about AUOF among our target audience (teachers and youth leaders aged 25–65). This approach directed traffic to our YouTube channel, Figshare and website, resulting in a rise of around 20–150 website visits within 2–4 days after our X (Twitter) posts.

### Wikipedia editing

In 2022 and 2023 we hosted 20 undergraduate students as part of the Summer Teams Enterprise Programme (STEP). The students were tasked with creating and updating articles connected to soil microbes on both English Wikipedia and Simple Wikipedia. Simple Wikipedia’s target audience are young people and those with English as a second language.

In 2023, Wikipedia training was expanded by including additional resources from WikiEdu to provide a more supportive and consistent training regime. As a result, 28.6 % students completed 6/6 of the training modules, 28.6 % completed 5/6, 21.4 % completed 4/6, and 28.6 % completed none of the training modules.

Overall, 20 articles were created from scratch and a further 39 articles were improved by editing as detailed in Table 3 below:

**Table 3.** Impact of STEP student editors on Wikipedia, as of 6 September 2023

Editor cohort(by year)	No. of editors	Articles created by the editors	Articles edited by the editors	Words added to all articles edited	References added to all articles edited	Subsequent views of edited articles by audience
**2022**	6	9	18	16 818	163	1284
**2023**	14	11	21	11 164	132	50 899
**All students**	20	20	39	27 982	295	52, 183

The new articles added to Wikipedia are listed in Table 4 below:

**Table 4.** New articles created from scratch and added to either Simple English Wikipedia or English Wikipedia by our editors, as indicated by an +. Bacterial genera and species are in italics. The remaining articles are primarily about antibiotics

Article title (A → Z)	Simple English Wikipedia	English Wikipedia
***Acidobacteria***	**+**	
**Azithromycin**	**+**	
***Azotobacter***	**+**	
***Caulobacter***	**+**	
**Cephalosporins**	**+**	
**Co-amoxiclav**	**+**	
**Daptomycin**	**+**	
***Enterobacter***	**+**	
***Enterococcus faecium***	**+**	
**Fluoroquinolones**	**+**	
***Glomeromycota***	**+**	
***Nitrobacter***	**+**	
***Nocardia***	**+**	
***Nocardioides***	**+**	
***Penicillium***	**+**	
***Planctomycetota***	**+**	
***Serratia***	**+**	
**Streptomycin**	**+**	
***Verrucomicrobia***	**+**	
**Unclassified *Bradyrhizobium***		**+**
***Variovirax***		**+**

The articles that already existed and were subsequently edited by our student editors are listed in Table 5 below:

**Table 5.** Existing articles edited on either Simple English Wikipedia or English Wikipedia by our editors, as indicated by an +. Bacterial genera and species are in italics. The remaining articles are primarily about antibiotics

Article title (A → Z)	Simple English Wikipedia	English Wikipedia
***Acinetobacter baumannii***	**+**	
***Ascomycota***	**+**	
***Bacteroidetes***	**+**	
***Bradyrhizobium***	**+**	
***Burkholderia***	**+**	
***Klebsiella pneumoniae***	**+**	
***Micromonospora***	**+**	
***Mycobacterium***	**+**	
***Nocardia***		**+**
***Pseudomonas***		**+**
***Pseudomonas aeruginosa***	**+**	
***Rhizobia***	**+**	
***Rhizoctonia***	**+**	
**Serotonin norepinephrine reuptake inhibitor**		**+**
***Streptomyces***	**+**	**+**
***Variovorax***		**+**

Improvements to articles can be measured using an artificial intelligence tool called Objective Revision Evaluation Service (ORES). ORES helps human editors to improve the quality of edits and articles on Wikipedia by predicting the quality of edits that are made, as well as the quality of the articles. The changes in the ORES scores for each article, pre- and post-edit, were visualised using a tool called Vega (https://vega.github.io/editor). Vega visualisations are interactive, dynamic, and designed to be viewed online. A static view for each article can be found in Fig. S1, available in the online version of this article.

### Evaluation

**Fig. 4.**
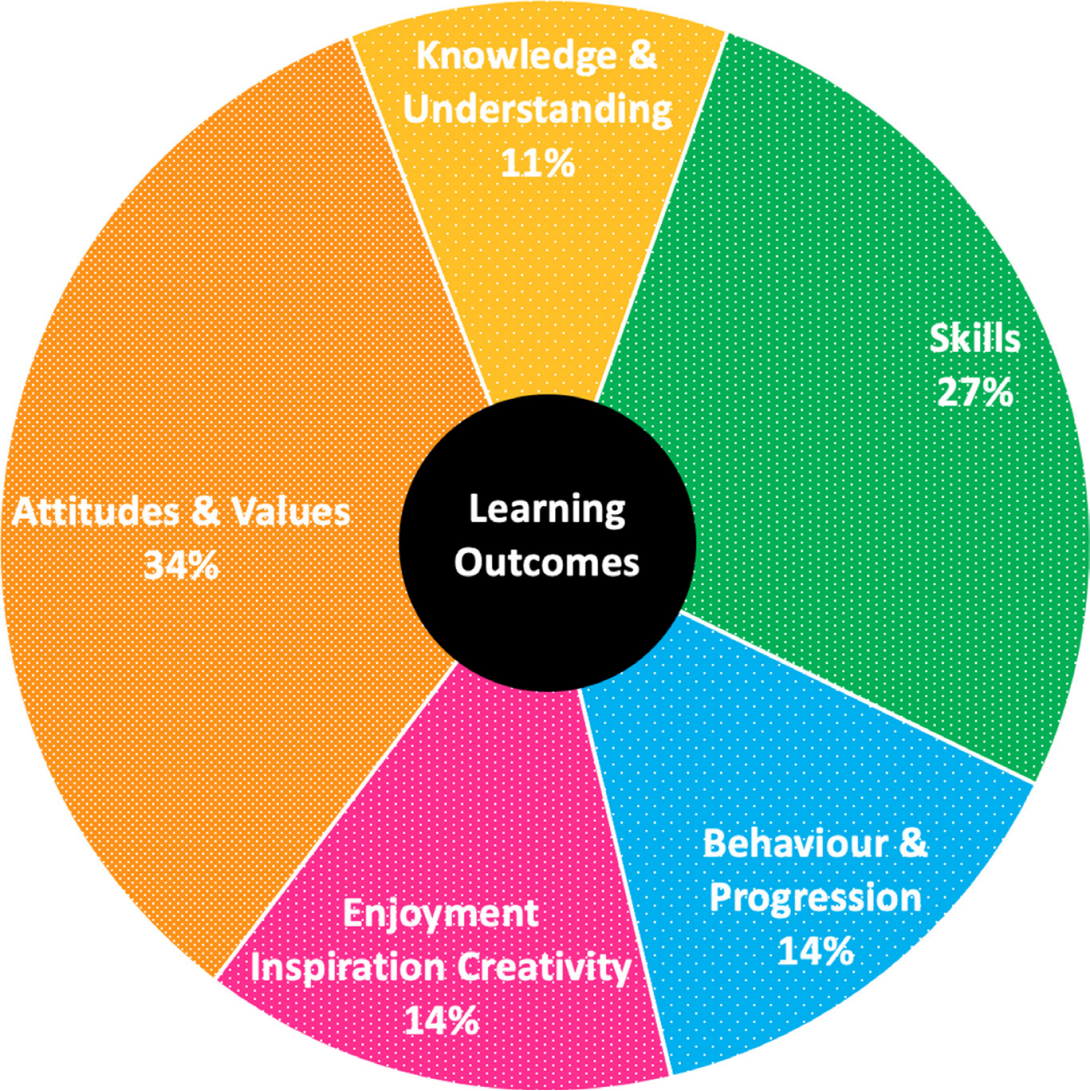
Range of learning outcomes achieved for undergraduate students on the STEP 2023 programme. Using Arts Council England Generic Learning Outcomes (GLO), we coded a total of 71 comments from seven undergraduates taking part in the STEP 2023 Wikipedia editing who received the expanded Wikipedia training.

Feedback from undergraduate students taking part on the STEP 2023 programme (all of whom are authors on this manuscript) was coded for common themes using the Arts Council England Generic Learning Outcomes Framework [77] (Fig. 4). This framework is designed to support the galleries, libraries, and museums sector (GLAM) in analysing qualitative feedback received from their visitors by breaking it down into five key themes.

## Discussion

Our main objective was to raise science capital and address the STEM skills gap through contribution to antibiotic discovery and awareness by engaging participants in a citizen science project. Focussing on the dimensions of science capital [42] AUOF raised learners' scientific literacy, helped explore science-related attitudes, values, and dispositions. Scientific literacy includes interpreting experimental findings, asking and seeking information to answer questions, and describing and explaining ideas. If we look across age groups, we have learners demonstrating scientific literacy in ways novel to them as individuals, for example entering data on our interactive map, asking us questions and talking about researchers' answers, interviewing us and editing a podcast, creating learning materials and videos, writing and editing Wikipedia articles. Through our learning materials and conversations, we stressed the importance of learners recognising that they were working like scientists, and we provided a variety of role-models within our teams when we produced YouTube and X (Twitter) content including our contribution to the Smashing Stereotypes campaign. Furthermore, AUOF deepened the understanding of science’s transferability, promoted recognition and application of science skills, fostered connections with individuals in science-related roles, and encouraged talking about science in daily life.

Reviewing our logic model, the assumption that all Fife primary schools would not be closed due to COVID-19 within the next two academic years proved to be inaccurate. Time availability became a significant challenge, especially for teachers adapting to online schooling. Because of COVID-19 disruptions, we lack enough pre- and post-intervention data to determine whether teachers and learners in our project showed low STEM confidence and science capital. During the first COVID-19 lockdown, our local community enthusiastically engaged in our consultation phase. Researchers and university students wanted to collaborate with teachers and learners to create resources matching the Curriculum for Excellence with potential for training using our digital materials. Teachers and learners showed interest in continuing dialogue with the University of St Andrews.

Lockdowns presented schools with challenges, reflected in the suboptimal response to our call. Our project was poised for broader implementation when the second lockdown began, prompting a strategic shift to focus on closer collaboration with fewer groups. This allowed for more sustained engagement, yielding unexpected outcomes such as the co-creation of a podcast [78] broadcast on Listen Lanarkshire.

In-person workshops would have been half-day or day visits however, with this model, schools and groups had the kit boxes for several weeks so could collect data and carry out activities over a longer period to suit their timetables and so engagement with the project was sustained for longer. The overall approach was more bottom-up with teachers and youth group leaders being more actively involved and learning about the science of the project than if led by an external visitor. The interactive map was designed and built by us to allow for remote data entry from participants whereas in the original plan we were considering outsourcing the website design and entering the data ourselves. This led to both participants and our project team gaining new skills. Switching to kit boxes extended engagement to learners at greater distance from the University of St Andrews, benefiting schools and youth groups amidst restricted STEM opportunities and broadening inclusion. We have donated our kit boxes to the STEM Ambassadors in Scotland resource hub [79] to ensure legacy. Our digital resources are widely shared, as shown by Figshare download metrics. Integrating these resources into a larger repository via our website enhances accessibility and their potential impact.

The pandemic posed difficulties for co-creation with teachers and pupils. Our project found alternative routes for collaboration mitigating these challenges. Through undergraduate interns and researchers, we produced videos directly answering primary school questions. As restrictions eased, iGEM students created videos on soil, Nuffield Research Placements students engaged in lab work and data analysis, and online/hybrid working led to impactful Wikipedia editing with STEP students during university holidays. This provided schools with specific data about the microbes in their samples, closing the loop from submission to feedback. Local secondary school students helped design and trial learning materials based on our data. After restrictions were lifted, we tested more in-person engagement activities. We had to be responsive to the ever-changing restrictions imposed by the COVID-19 pandemic. If AUOF was repeated or reinstated in the future, we would introduce routine before and after evaluations, and offer further twilight training sessions for teacher and youth group leaders at all stages of the core learning pathway.

Multi-level educational engagement brought advantages, bridging the comprehension gap among researchers, university students, secondary, and primary audiences, especially in simplifying terminology and explaining complex ideas. Secondary and undergraduate level learners, more familiar with school teaching styles, helped with the design of suitable educational material enhancing overall understanding of the Curriculum for Excellence.

## Conclusions and reflections

*‘*It’s important that young people know about antibiotic resistance. It’s good to be able to explain the facts of antibiotic resistance to them, why you are doing the project and that this is kind of how researchers are kind of trying to overcome the problem.’

*AUOF Participant*

We involved learners in Learning for Sustainability, combining outdoor soil microbe education and citizen science to tackle antibiotic resistance and foster global citizenship. Our project allowed participants to enhance their science capital by improving scientific literacy, understanding practical science applications, connecting with individuals in science-related roles, and promoting discussions about science in daily life.

Capitalising on COVID-19 opportunities, AUOF engaged learners from primary to postgraduate levels, adapting to pandemic challenges. Kit boxes and a hybrid model extended outreach, continuing post-pandemic. Every participant, regardless of age or expertise, was an essential part of the extended team, promoting two-way learning.

## supplementary material

10.1099/acmi.0.000710.v3 Uncited Supplementary Material 1.

## Abbreviations

AMR: antimicrobial resistance
AUOF: antibiotics under our feet
CfE: curriculum for excellence
GLAM: galleries, libraries, and museums sector
GLO: generic learning outcomes
JHI: James Hutton Institute
ORES: objective revision evaluation service
ScotPEN: The Scottish Public Engagement Network
S.T.A.F.F.: St Andrews flexible fundinternships
STEM: science, technology, engineeringand mathematics
STEP: Summer Teams Enterprise Programme
SWEA: ScotPEN Wellcome Engagement Award
