# Supplementary material for: Unearthing new learning opportunities: adapting and innovating through the ‘Antibiotics under our feet’ citizen science project in Scotland during COVID-19
Source: Access Microbiol. 2024 Jun 19;6(6):000710.v3. doi: 10.1099/acmi.0.000710.v3 (PMC11261732; doi:10.1099/acmi.0.000710.v3)
Supplement: Uncited Supplementary Material 1. [file acmi-6-00710-s001.pdf]

Supplementary information for

Unearthing New Learning Opportunities: Adapting and Innovating through the "Antibiotics Under Our Feet" Citizen Science Project in Scotland during COVID-19

Authors:
Rebecca Cornwell[1], Kirsty Ross [2], Caius Gibeily[3], Isobel Guthrie[3], Pak Hei Li[3], Laurence Taylor Seeley[3], Yaxuan Kong[3], Ava True[3], Arun Barnes[3], Emma Nimmo[3], Gloriya Len[3], Ioana Oprea[3], Boyang Lin[3], Aswin Sasi[4], Vicky Chu[3], Chloe Davidson[3], Daniil Ulasavets[3], Grace Renouf-Bilanski[3], Maria Dmitrieva[3], Yana Leung[3], Ziying Ye[3], Sasha Brown[3], Meghna Vaidya[3], Jenna Hynes[3], Catherine Mullner[3], Priyansha Agarwal[3], Paul Johnston[5], Charlotte Thorley[6], and Clarissa Melo Czekster[1]

Affiliations:

[1] University of St Andrews, School of Biology, North Haugh, St Andrews, KY16 9ST, UK.
[2] University of St Andrews, School of Computer Science, North Haugh, St Andrews, KY16 9SX, UK.
[3] University of St Andrews, University of St Andrews, College Gate, St Andrews, KY16 9AJ
[4] University of St Andrews, School of Physics and Astronomy, North Haugh. St Andrews, KY16 9SS, UK
[5] University of St Andrews, School of Medicine, North Haugh, St Andrews, KY16 9TF
[6] Independent researcher

Corresponding Author
Clarissa Melo Czekster - School of Biology, Biomedical Sciences Research Complex, University of St Andrews, St Andrews, Fife KY16 9ST, U.K
ORCID https://orcid.org/0000-0002-7163-4057; Email: cmc27@st-andrews.ac.uk

Supplementary methods:

DNA extraction

DNA was extracted following the instructions detailed on the "Qiagen DNeasy PowerSoil Pro" DNA extraction kit (Cat # 47014). All steps were carried out as instructed by manufacturer including the optional 5 minute incubation steps on ice. DNA was eluted in 100uL sterile H_2O and kept in the -20 °C for further analysis.

Quality Control

All extracted DNA samples were quantified using a DeNovix® DS-11 FX Spectrophotometer to determine the concentration, Abs260/Abs230, Abs260/Abs280 and Abs260 values. DNA integrity was accessed by a Qubit Assay using an adapted BMG LabTech Microplate Reader[1]. A standard curve was generated with 190µL working solution + 10 µL Standard (0 ng/µL or 10ng/µL of DNA). 1% TAE agarose gels were also ran with 5 µL each DNA sample, to verify genomic DNA was not degraded.

DNA next generation sequencing

DNA samples were sent for Illumina Sequencing at Eurofins or MicrobeNG using Illumina NovaSeq. For samples sequenced by Eurofins, raw sequence data in addition to information on bacterial diversity and abundance, the presence of antibiotic resistance genes and functional genes were provided. For samples sequenced by MicrobeNG, raw sequence data in addition to information on bacterial diversity and abundance were obtained.

Supplementary Figure 1 Histogram plots of changes in ORES scores on each article from STEP 2023 (pre-editing in purple and post-editing in green). Those articles that start at zero were created by STEP undergraduates. Articles where the two peaks overlap are usually well-developed articles with little room for improvement. A shift to the right indicates that the article has been improved, receiving a higher ORES score than the original article.

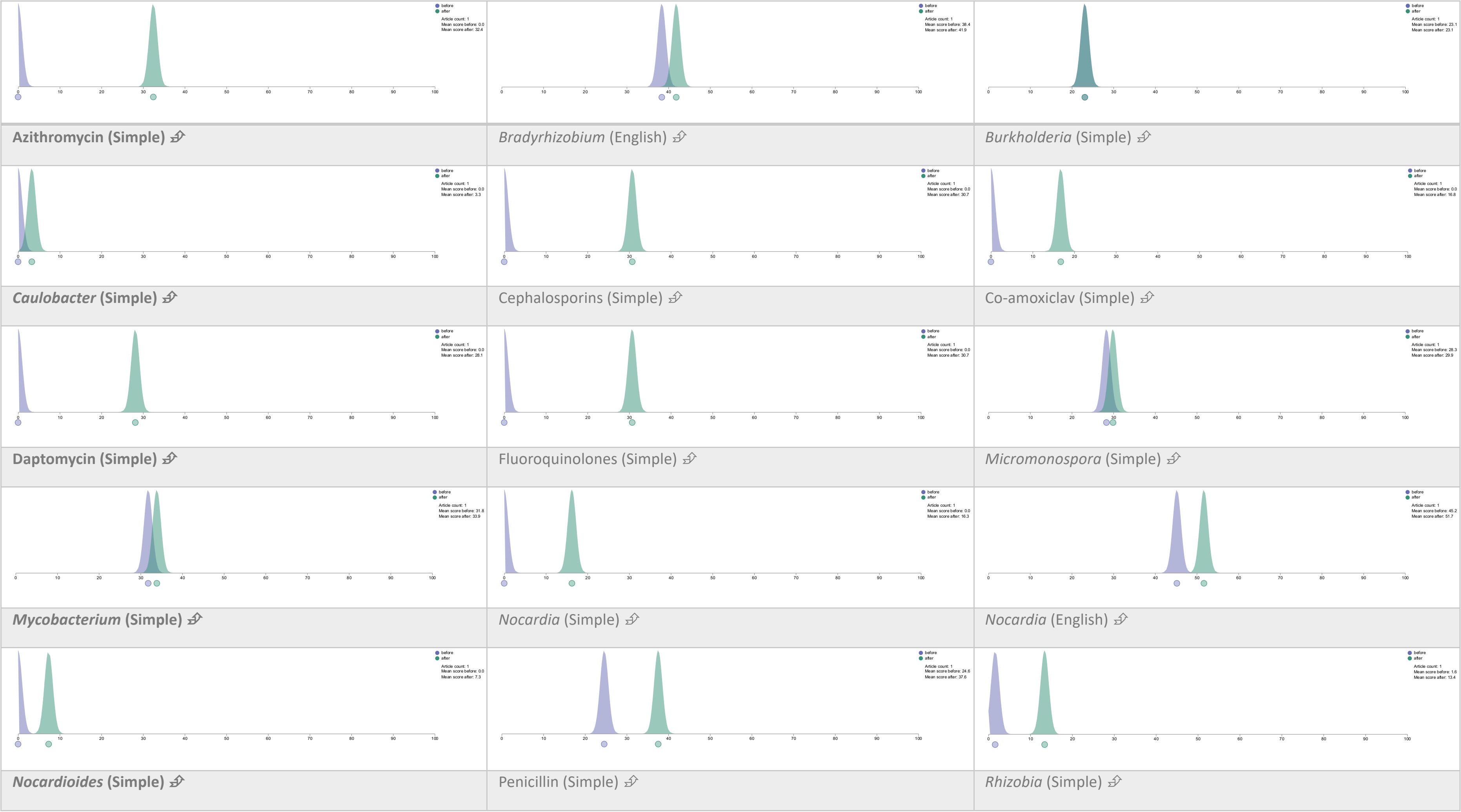

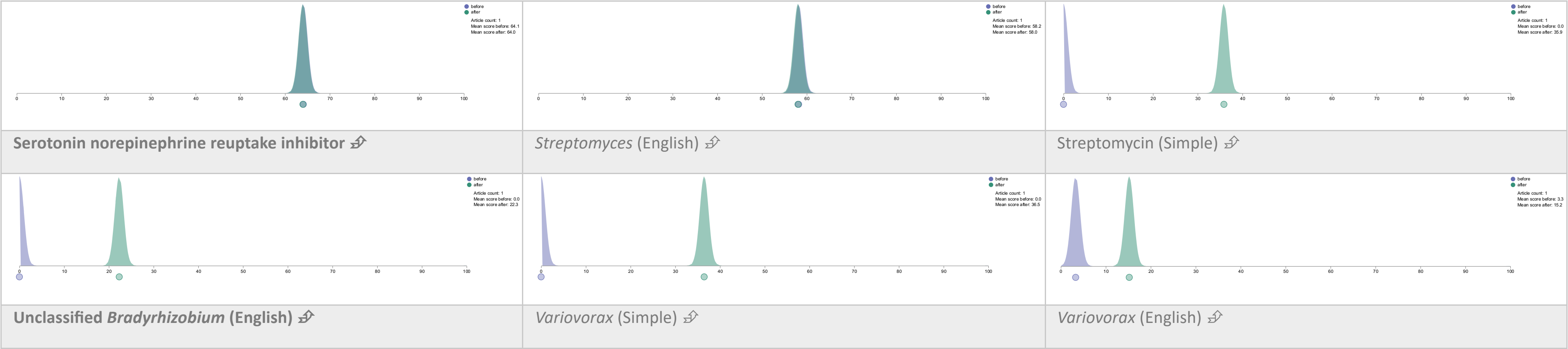

before
after
Article count: 1
Mean score before: 64.1
Mean score after: 64.0
Serotonin norepinephrine reuptake inhibitor
before
after
Article count: 1
Mean score before: 58.2
Mean score after: 58.0
Streptomyces (English)
before
after
Article count: 1
Mean score before: 0.0
Mean score after: 35.9
Streptomycin (Simple)
before
after
Article count: 1
Mean score before: 0.0
Mean score after: 22.3
Unclassified Bradyrhizobium (English)
before
after
Article count: 1
Mean score before: 0.0
Mean score after: 36.5
Variovorax (Simple)
before
after
Article count: 1
Mean score before: 3.3
Mean score after: 15.2
Variovorax (English)
0 10 20 30 40 50 60 70 80 90 100
